# Donor shortage in heart transplantation: How can we overcome this challenge?

**DOI:** 10.3389/fcvm.2022.1001002

**Published:** 2022-10-17

**Authors:** Matteo Cameli, Maria Concetta Pastore, Alessandro Campora, Matteo Lisi, Giulia Elena Mandoli

**Affiliations:** ^1^Division of Cardiology, Department of Medical Biotechnologies, University of Siena, Siena, Italy; ^2^Santa Maria delle Croci Hospital, Ravenna, Italy

**Keywords:** heart transplantation (HT), heart failure, heart donor, ADONHERS protocol, mechanical circulatory support

## Introduction

Heart transplantation (HT) is the treatment of choice for carefully selected patients with advanced or end-stage heart failure (HF) in the absence of contraindications (1) (Table 1) with an overall median survival of 12.5 years and conditional survival of 14.8 years for those who surviving after the first year (3).

**Table 1:** Heart transplantation: Indications and contraindications.

**Indications**
Advanced HF (2)
No other therapeutic option, except for LVAD as BTT
**Contraindications**
*Assolute*
Active infection^a^
Severe peripheral arterial or cerebrovascular disease
Pharmacologic irreversible pulmonary hypertension (LVAD should be considered to reverse elevated pulmonary vascular resistance with subsequent re-evaluation to establish candidacy)
Malignancy with poor prognosis (a collaboration with oncology specialists should occur to stratify each patient as regards their risk of tumor progression or recurrence which increases with the use of immunosuppression)
Irreversible liver dysfunction (cirrhosis) or irreversible renal dysfunction (e.g., creatinine clearance <30 mL/min/1.73 m^2^). Combined heart-liver or heart-kidney transplant may be considered
Systemic disease with multiorgan involvement
Other serious comorbidity with poor prognosis
Pre-transplant BMI >35 kg/m^2^ (weight loss is recommended to achieve a BMI <35 kg/m^2^)
Current alcohol or drug abuse
Psychological instability that jeopardizes proper follow-up and intensive therapeutic regime after heart transplantation
Insufficient social supports to achieve compliant care in the outpatient setting
Relative
Age > 65 years
Obesity (BMI between 30 and 35 kg/m^2^)
Cachexia
Irreversible chronic renal failure (clearance <30 ml/min) (except for combined transplant)
Reduced individual compliance and/or poor family support
Diabetes with organ damage (except for non-proliferative retinopathy) or with low glycemic control (HbA1c >7.5 mg/dl or 58 mmol/mol)
Smoking habit (suspension required for at least 6 months)
HCV and/or HBV-related chronic hepatopathy
Severe osteoporosis
Chronic pulmonary disease with severe functional and morphological alterations (GOLD classification)
Severe chronic peripheral vasculopathy based on imaging tests
HIV infection (Useful opinion of the infectious disease specialist)

BMI, body mass index; BTT, bridge to transplantation; HF, heart failure; LVAD, left ventricular assist device.

a Active infection is a relative contraindication to transplant although in some cases of infected LVADs it may actually be an indication. Adapted from Crespo-Leiro et al. (2).

According to the Global Observatory on Donation and Transplantation, at the end of 2020 (during the COVID-19 pandemic), 2081 patients underwent HT across all European Union countries. In the same year, subjects on the active waiting list were 6,352, among which 435 (7%) died while waiting for a suitable donor (2). Data from 2019 were comparable: 2,269 patients underwent HT, 6,940 subject were on the waiting list, among which 481 (7%) died (4).

Over the past two decades, we registered an increase of 35% in HTs per million population (PMP) rate in Europe, with an annual percentage change (APC) of 1.4% [95% CI (1.1–1.7), *P* < 0.0001]. The increase was particularly relevant in Central Europe, where HTs PMP rate raised from 0.65 in 2000 to 2.93 in 2019 {APC 9.9% [95% CI (8.1–11.8), *P* < 0.0001]} and in Northern Europe, PMP rate from 2.97 in 2000 to 5.18 in 2019 {APC 2.7% [95% CI (1.8–3.7), *P* < 0.0001]} (5).

Despite the reached HT rates, the demand for HTs is persistently higher than the current offer, mainly due to the aging of the global population, the improved overall survival after a myocardial infarction and the better outcome of patients with HF. Solving the current shortage of donor hearts is a major issue, involving medical, legal, religious, cultural, and ethical considerations. This requires combined efforts to expand currently accepted and new selection strategies but also to improve alternative strategies to transplantation. In this editorial, we provide a practical review of selected contemporary advances and challenges in this field.

## Increasing the pool of hearts for transplantation with currently accepted methods

The standard cardiac donor selection criteria are listed in Table 2.

**Table 2:** Traditional cardiac donor selection criteria.

**Traditional cardiac donor selection criteria**
Age <55 years old No history of chest trauma or cardiac disease No prolonged hypotension or hypoxemia Appropriate hemodynamics Mean arterial pressure >60 mmHg Central venous pressure 8 to 12 mmHg Inotropic support <10 mg/kg/min (dopamine or dobutamine) Normal electrocardiogram Normal echocardiogram Normal cardiac angiography (if indicated by donor age and history Negative serology (hepatitis B surface antigen, hepatitis C virus and human immunodeficiency virus)

Adapted from Sellke et al. (6).

Despite strenuous political efforts to promote organ donation and implement donors selection strategies, a lot of differences persists across Europe and other Continents. Geography should not represent a determinant of the possibility of a potential candidate to receive HT, but the disparities in HTs rates among European countries suggest many factors needing improvements, including: optimization and coordination of the donation process, education of professionals, patients and the general population, disposal of appropriate financial and legal frameworks. Coordinating the donation process and expanding the criteria for donation are primary elements. Other factors, such as benchmarking, research, and efforts to overcome inequities, might not directly affect the number of HTs, but remain pivotal for ethical reasons and as support for further strategies. Adjustments in the allocation policy have been developed to address these issues, but disparities have not been solved yet. Broader organ sharing was introduced in the national allocation systems for heart, lung, and more recently liver and kidney to reduce geographical differences. However, it is still unclear whether these policy adjustments will eliminate geographical disparities (7).

### Age

An effective way to solve the current shortage of donors would be an upward shift of the donor age cut-off limit (from the current 55–65 years). Age-related high prevalence of asymptomatic coronary artery disease and cardiomyopathy severely limit the feasibility of this approach unless a functional screening on the candidate donor heart is performed (8, 9). Although older donors are associated with higher recipient mortality risk, favorable survival has been shown with organs from donors >50 or even >60 years old (10).

For instance, in Italy, about 300 HTs are performed each year with more than 800 patients on the HT list. Over a total of about 1,200/year donor pool, 600 donors are aged <55 years, and 300 of them are eligible for heart donation; since 600 potential donors are aged >55 years, the recruitment of even 25% of the currently dismissed aged donor pool would thereby dramatically decrease the current donor supply shortage (11, 12).

### ADONHERS protocol

**Figure 1:**
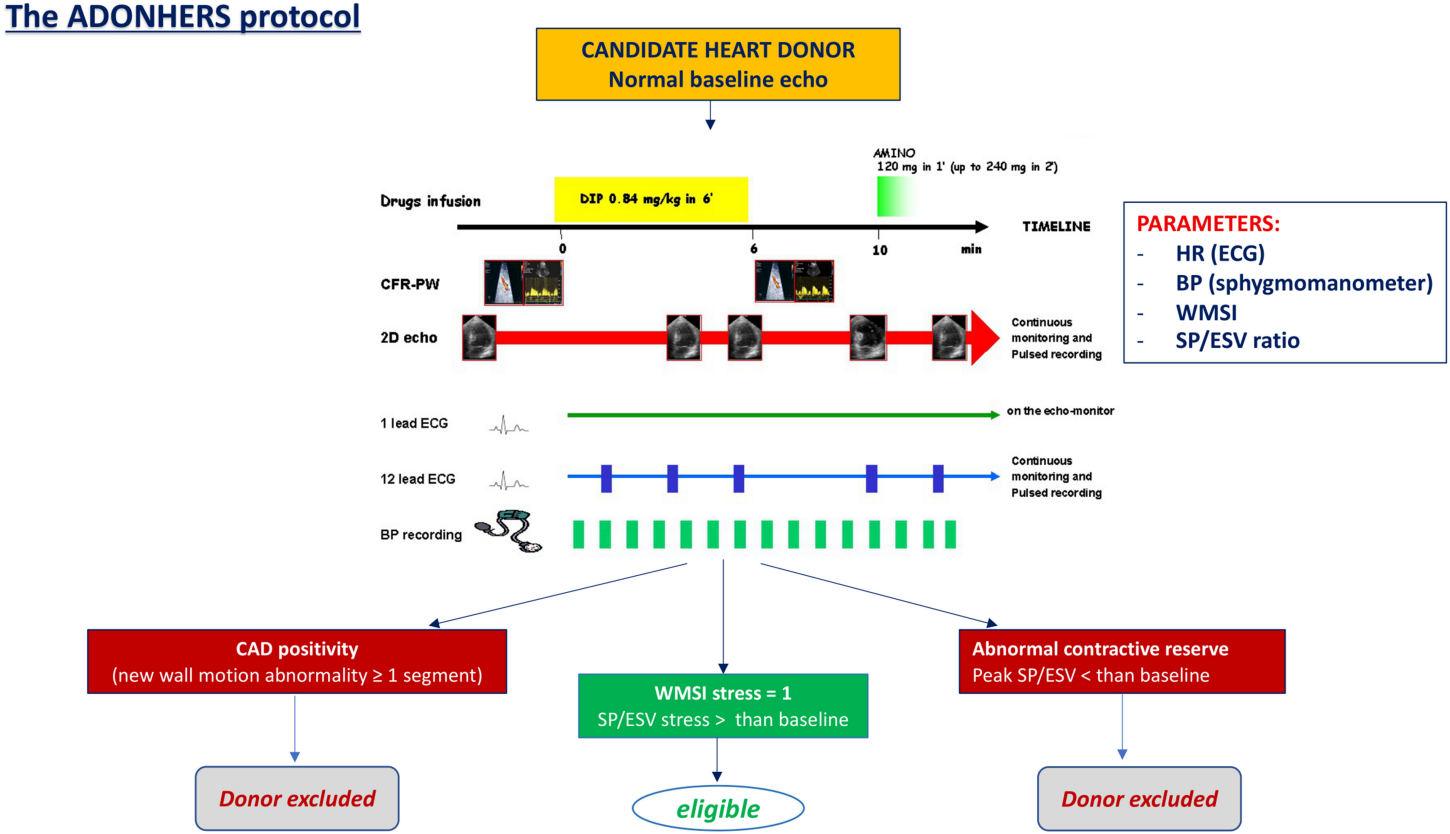
The dipyridamole stress echo in the Adonhers protocol [adapted from Franchi et al. (14)]. When resting echocardiography was normal a pharmacological stress echo test was performed using dipyridamole (0.84 mg/kg in 6 min). Three criteria of stress echo positivity were accepted a priori, excluding the heart from eligibility for donorship: (1) Regional wall motion abnormalities at rest or during stress. (2) A LV elastance falling during stress. (3) A submaximal stress halted due to non-diagnostic limiting effects before completion of the infusion, since a submaximal test dramatically lessens diagnostic and prognostic power. Accepting a heart was done in conformity with clinical and emergency criteria in use. BP, blood pressure; CAD, coronary artery disease; ECG, electrocardiogram; HR, heart rate; SP/ESV, systolic pressure/end systolic ratio; WMSI, wall motion score index.

The ADONHERS protocol has been developed in Italy (first in Emilia-Romagna and Tuscany) with the background of a possible donors age extension (from 55 to 65 years old) after an accurate screening by stress echocardiography to exclude subtle coronary artery disease (13). After excluding global o regional wall motion abnormalities, severe LV hypertrophy, severe valve heart diseases, the brain-dead potential donor, aged >55 years old or <55 years with known multiple cardiovascular risk factors, undergoes dipyridamole stress echocardiography. Inducible ischemia identified by new LV wall motion abnormalities and/or LV abnormal contractile reserve (by Sagawa index) excludes the patient from donation. On the other hand, LV preserved contractile function and normal global and regional function determine marginal donor eligibility (Figure 1) (15). One of the possible limitations of stress echocardiography is the operator dependency. Speckle-tracking echocardiography, offering a quantitative objective analysis of myocardial deformation, may help to overcome this limit, and has showed an excellent feasibility in the analysis of LV myocardial longitudinal deformation both at baseline and peak stress in marginal heart donors. It may be used as a valuable additional mean to better interpret stress echocardiography results in marginal donors (16).

Donor comorbidities, such as diabetes mellitus and hypertension, might affect post-transplant outcomes. Such hearts are now used after excluding irreversible structural cardiac abnormality, and risk scores have been developed to help clinicians in these complex decisions and to guide donors' selection procedures (17, 18).

### HCV prophylaxis

Drug abuse in the community raised in the last years resulting in an increased number of organ donors died of overdose. Post-transplant survival in recipients of such organs seems favorable (19). Curative therapies for Hepatitis C virus (HCV) infection led to the use of HCV-positive donors for organ transplantation (20). An early prophylaxis strategy is correlated to low levels of viral transmission and avoids the development of HCV infection in the recipient, with a shorter duration of therapy and lower costs. In the first year after HX, outcomes of hearts from HCV-positive donors are similar to HCV-negative donors, although longer-term outcomes pertaining immunological activation, allograft rejection, and cardiac allograft vasculopathy remain uncertain (21).

### Long-term mechanical circulatory support

Long-term mechanical circulatory support (MCS) might represent an additional strategy to reduce organ shortage. In fact, a wider use of MCS has been described in patients with end-stage HF who show temporary relative contraindications for HT. Current indications for mid-term and long-term MCS include “bridge to transplantation,” BTT (patients with severe hemodynamic compromise on a HT waiting list), “bridge to recovery,” BTR (recently severe reduction of myocardial function with possible recovery), “bridge to decision,” BTD and “bridge to candidacy,” BTC (while defining the best management for end stage HF patient). The use of the available different devices should be individualized according to patient characteristics.

Mechanical ventricular assist devices were developed for the left ventricle (LVADs), for the right ventricle (RVADs) or as a full heart replacement. MCS can be divided into short-term and long-term devices. One of the major issue concerning LVAD implantation in the high post operatory mortality rate. In the EUROMACS registry, approximately one out of five patients died within 90 days after LVAD implantation and early mortality was primarily driven by multiorgan failure, followed by sepsis (22, 23).

In the same registry, outcomes of patients receiving MCS were reviewed from January 2011 to June 2020. Totally, 4,834 procedures in 4,486 individual patients (72 hospitals) were included, with a median follow-up of 1.1 (interquartile range: 0.3–2.6) years. During this timeframe, the annual number of implants (range: 346–600) did not significantly change (*P* = 0.41). Two thousands and thirty-six patients died, with an estimated mortality probability of 30.0, 44.5, and 55.5% at 1, 3 and 5 years, respectively. Survival rate was significantly different across different INTERMACS classes, eras, devices, and strategies. Eight hundred and sixty-four patients were successfully given transplants, with a probability of receiving a transplantation of 7.5, 20.2, and 25.2% at 1, 3 and 5 years, respectively. Eleven patients, originally listed as destination therapy (DT), received a transplant while 3 patients were weaned.

Comparative studies on the impact of LVAD implantation on clinical outcome as a BTT are lacking, being difficult to randomize an outpatient, candidate to HT, to a double surgical step (LVAD and HT) vs. a direct HT. However, the available evidence show that LVAD use for BTT guarantees excellent survival and a similar quality of life to that of patients undergoing direct HT. In particular, heart transplanted patients after BTT have a slightly higher mortality rate within 90 days from HT than patients directly sent to HT, but this difference would be probably compensated by the live-years gained using MCS as BTT rather than remaining on the waiting list without advanced therapy (23).

## Increasing the pool of hearts for transplantation with new methods

A lot of efforts are currently being invested in the improvement of the current methodologies and in the possibility of exploiting new technologies, to ensure better safety in conservation and transport of the donated organ. Promising data are emerging on the possibility of performing donation after circulatory death (DCD) and another interesting branch of research, which could be fundamental in solving the problem of the shortage of donors, is *xenotransplantation*.

### *Ex-vivo* heart perfusion

In particular, another way to expand the donor pool would be to remove the geographic constraints of ischemic time. This could be obtained with an *ex vivo* heart perfusion platform that maintains the donor heart in a warm, beating state for transplantation. Some small registries have demonstrated the safety of this procedure (24). In the largest randomized trial 130 patients were randomized to receive donor hearts preserved by using either the Organ Care System or standard cold storage. No differences were found in 30-day patient and graft survival rates or serious adverse events (25). The *ex vivo* perfusion platform offers great potential for extended criteria donor hearts, where cold storage would conventionally be associated with poorer outcomes.

### Donation after circulatory death

In the last few years, in order to solve the donor shortage, DCD has been studied for HT (26). In DCD, retrieval of hearts for transplantation occurs from patients whose death is declared and confirmed using cardiorespiratory criteria as life support is withdrawn. Particularly, these patients have severe, not reversible, brain damage, that does not meet brain dead criteria. The heart is removed and then, using *ex vivo* perfusion, resuscitated. The major challenges with HT from DCD are the minimization of ischemic injury of the donor organs, and the after-death assessment of myocardial viability, since, in the DCD, the heart is subjected to an unavoidable period of severe, warm ischemia. Nevertheless, in almost 50 DCD HT performed, post-HT survival and graft function to date seems to be comparable to those observed in contemporary HT performed with donation after brain death (DBD) (27–29).

These outcomes seem to be supported in recent single-center retrospective cohort study. This study analyzed right heart catheterization measurements, inotrope scores, echocardiograms, and clinical outcomes between DCD and DBD heart recipients. Forty-seven DCD and 166 DBD hearts were transplanted. Despite an early significant right heart function impairment in the DCD heart recipient group, the right heart function was similar in the two groups after 3 weeks from HT. Mortality was similar at 30 days (DCD 0 vs. standard 2%; *P* = 0.29) and 1 year post-HT (DCD 3% vs. standard 8%; *P* = 0.16) (30).

### Xenotransplantation

Xenotransplantation has required, and is still requiring, scientific advances to overcome challenges of evolutionary distance between species, transmission of zoonosis into the human pool, immunological barriers that cause hyperacute rejection, allograft failure due to thrombotic microangiopathy, and, moreover, it raises ethical concerns of distributive justice. On January 7th, 2022, a successful genetically edited porcine to human HT was performed with 60 days patient survival (31). On autopsy, the xenograft showed findings that were not consistent with typical rejection: it was edematous, nearly doubled in weight, and histologic examination revealed scattered myocyte necrosis, interstitial edema, and red-cell extravasation, without evidence of microvascular thrombosis. Studies are currently under way to identify the mechanisms responsible for these changes (32) and clearly, this experience has highlighted the presence of pathophysiological patterns that have yet to be understood. The topic of xenotransplantation, which could allow new, exciting therapeutic perspectives, raises political, ethical and moral concerns, that will have to be addressed in the years to come. Nevertheless, it represents an interesting new frontier which could make a significant contribution in solving the problem of donor shortage, guaranteeing survival hopes for patients with terminal heart disease currently lacking therapeutic alternatives.

## Alternative therapeutic strategies to transplantation

Although HT represents the “gold standard” treatment for patients with advanced HF mainly due to its results in terms of prolongation of life expectancy, competitive technologies are currently under examination as valid therapeutic strategies. The efforts are mainly directed toward long term MCS, such as LVAD and fully implantable mechanical assist systems, or new cutting-edge technologies, such as gene therapy and tissue engineering (33).

### Left ventricular assist devices

The most used devices for long-term MCS are those supporting the left ventricle. However, they are burdened by socioeconomic limitations and complications. An accurate selection of candidate by multimodal imaging and right heart catheterization is mandatory to avoid post-implantation right ventricular failure. In particular, an echocardiographic evaluation plays a pivotal role in the evaluation of the patient before, during and after LVAD implantation in the attempt to exclude contraindications (Table 3), guide the implant, optimize pump settings according to patients' hemodynamic profile and exclude complications.

**Table 3:** General evaluation in the patient candidate for left ventricular assist devices.

	**Factors conditioning the LVAD implant**	**Contraindications**
Age		>75 years
Life style		Smoking habit, addiction to alcohol and psychotropic substances
Non-cardiological conditions	Multiorgan dysfunction Neurological/psychiatric conditions Coagulation Kidney function Hepatic function Respiratory pathology Oncological pathologies Diabetes mellitus Obesity	Irreversible multi-organ failure Degenerative neuro / muscular diseases Recent stroke Psychiatric/cognitive and / or psycho-social conditions with poor adherence to treatment Coagulopathies Uncontrollable bleeding Irreversible renal failure Severe hepatic insufficiency Severe respiratory failure (FEV1 <50%) Life expectancy <2 years Poor control of blood glucosevalues
Cardiological conditions	Left ventricle - Recent myocardial infarction - Left endoventricularthrombosis	Apical infarction
	Right ventricle - Right ventricular function (multiparametric approach) and pulmonarypressures	Severe right ventricular dysfunction Pulmonary hypertension
	Valvulopathies and valve prostheses - Aortic valve - Mitral stenosis - Tricuspid insufficiency - Endocarditis	Uncorrectable moderate-severe insufficiency Uncorrectable moderate-severe insufficiency Active endocarditis
	Arrhythmias - Atrial tachyarrhythmias with a high response rate - Ventriculartachycardias	Uncontrolled ventricular tachyarrhythmias
	- Previous cardiacsurgery	Previous ventriculoplasty
	Other cardiovascularconditions - Atrial and interventricular septal defects - Restrictive or constricting forms - Anomalies affecting the ascending aorta - (dilation, calcifications, atherosclerotic plaques) - Peripheral vasculopathy - Congenital heartdisease	

FEV1, forced expiratory volume in the firstsecond.

In patients undergoing LVAD implantation, the main long-term complications include infective complications, bleeding and cerebrovascular complications of both ischemic and hemorrhagic nature. In addition, malfunctioning of the LVAD, worsening of aortic regurgitation, ventricular arrhythmias and pump thrombosis may occur. Right ventricular (RV) failure however has however the main relevance on survival. preoperative RV dysfunction should be exclude due to the incapability of the right RV to support the newly increased systemic flow after the implantation. Clinical, echocardiographic, and hemodynamic predictors have been studied but current algorithms for post-LVAD RV failure risk prediction only modestly perform when applied to external populations (34). Recent promising preoperative laboratory and echocardiographic predictors are emerging, like pulmonary artery pulsatility index (PAPi), N-terminal pro brain natriuretic peptide (NT-proBNP) and free wall RV longitudinal strain (fwRVLS) by speckle tracking echocardiography; the latter, representing intrinsic RV myocardial deformation, was the strongest independent predictor of post-LVAD RV failure (35, 36).

The development of durable right-sided mechanical support would improve treatment of patients with RV failure and fully implantable mechanical assist total heart systems, will undoubtedly provide new options for our patients in the future (37, 38).

### Total artificial heart

A total artificial heart (TAH) has so far been successfully implanted in over 1,700 patients as a temporary life-saving technology as a BTT (39).

However, after more than six decades of research on TAHs, a device suitable for DT is not yet available. The high rate of complications, bulky devices, poor durability and biocompatibility and low patient quality of life, are some of the main issues limiting TAH. Promising perspective that could help to overcome these limitations are emerging, thanks to the quick developing of innovations in battery technology, wireless energy transmission, biocompatible materials, and soft robotics.

Innovations in the field of biological therapies are leading to bioartificial heart developing, again as possible candidate to overcome shortage of donor hearts. Many intuitions derived from TAHs can also be applied in projecting a bioartificial heart. However, there are some features that are unique to a bioartificial organ, such as the use of cells as an energy source, the necessity of vascularization and the capability of endogenous repair. Many efforts have been addressed on finding cell sources and a suitable vascularized scaffold and now, with the development of inducible pluripotent stem cell technology, autologous tissue engineering is conceivable. It is an exciting era for biomedical engineering, which carries great potential in addressing damaged organs. That could be done either *via* repair or replacement and the development in heart bioengineering have been astounding. However, further research still needs to be run to provide a mechanically, electrically, and physiologically well-coordinated organ and, ultimately, to successfully transplant it into patients. A coordinated approach between researchers, clinicians, regulatory bodies and society should be promoted to develop unlimited immunotolerant grafts.

### Gene therapy

Another innovative therapeutic option is gene therapy. This treatment alters the genetic content of cells to modify target organ therapeutic protein or RNA expression. It has already been successfully introduced into clinical practice for the treatment of various diseases. The greatest benefit of its use in HT would probably be in the prevention of post-transplantation complications, such as primary graft dysfunction, cardiac allograft vasculopathy, and rejection. Additionally, gene therapy can be used to minimize and, potentially, eliminate the need for post-HT immunosuppression. Over the years, researchers have designed and developed several animal models and delivery techniques, with the aim of achieving strong gene expression in the heart. However, none of these methods has been so far successfully translated into clinical practice (40). The recent advances in *ex vivo* perfusion for organ preservation may provide potential ways to overcome many of the barriers that are currently preventing this method from entering clinical practice in HT. Optimizing vector selection for gene-carrying and delivery, and the selection of the therapeutic gene to be conferred are also a key point for implementing gene therapy in HT.

## Conclusions

**Figure 2:**
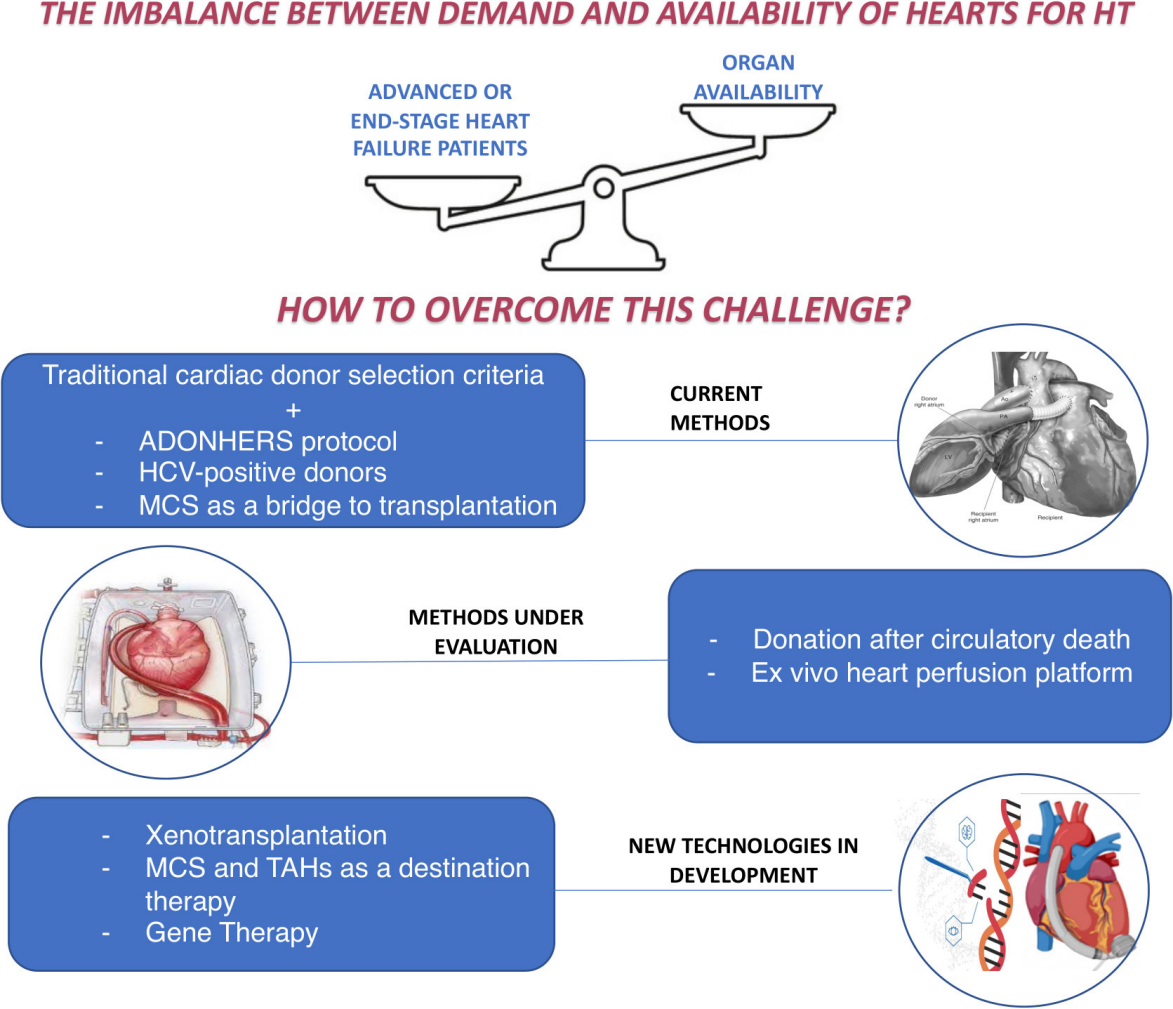
How to overcome the imbalance between demand and availability of hearts for heart transplantation: current methods and new perspectives. ADONHERS, aged donor heart rescue by stress-echo; HCV, Hepatitis C virus; HT, Heart Transplantation; MCS, mechanical circulatory support; TAHs, Total artificial hearts.

The issue of the imbalance between the demand and the offer of hearts for HT remains a therapeutic obstacle in advanced HF, whose incidence is continuously growing. In the last decades, many options were proposed to improve the selection of candidates, the survival of patients on the waiting list and the number of available donors, as well as to guarantee alternatives in non-eligible patients (Figure 2).

Reasonably, optimization and coordination of the donation process and an improved management of the currently available methods, would not allow to completely overcome the gap between heart demand and availability. However, it could compensate the difficulties in the treatment of the most critical patients and improve their overall survival.

HT, and transplants in general, remains a complex topic, not only involving clinicians, but also political, economic, and ethical issues. The quick and continuous technological advances could provide new therapeutic alternatives in the near future; however, it would be essential to overcome the obstacles limiting the availability of hearts for transplantation with the means already at our disposal. Certainly, promoting education and raising awareness of the society, greater political commitment and improving international collaboration have a fundamental role in this direction.

## Author contributions

MC and MCP contributed to the conception of the manuscript. MCP and AC performed the data research and analysis. MC, MCP, and AC drafted the manuscript. ML and GM contributed to manuscript revision and editing. All authors read and approved the submitted version.

## Conflict of interest

The authors declare that the research was conducted in the absence of any commercial or financial relationships that could be construed as a potential conflict of interest.

## Publisher's note

All claims expressed in this article are solely those of the authors and do not necessarily represent those of their affiliated organizations, or those of the publisher, the editors and the reviewers. Any product that may be evaluated in this article, or claim that may be made by its manufacturer, is not guaranteed or endorsed by the publisher.
